# Biological Aging and the Motoric Cognitive Risk Syndrome

**DOI:** 10.1002/alz.089384

**Published:** 2025-01-09

**Authors:** Sanish Sathyan, Hamilton Se‐Hwee Oh, Ayers Emmeline, Jarod Rutledge, Dristi Adhikari, Tina Gao, Sofiya Milman, Tony Wyss‐Coray, Nir Barzilai, Joe Verghese

**Affiliations:** ^1^ Albert Einstein College of Medicine, Bronx, NY USA; ^2^ Wu Tsai Neurosciences Institute, Stanford University, Stanford, CA USA; ^3^ Department of neurology, Division of Cognitive and Motor Aging, Albert Einstein College of Medicine, New York, NY USA; ^4^ Department of Neurology, Division of Cognitive and Motor Aging, Albert Einstein College of Medicine, New York, NY USA

## Abstract

**Background:**

Motoric Cognitive Risk (MCR) syndrome is a predementia syndrome characterized by slow gait and subjective cognitive concerns. Individuals with MCR are at high risk of transitioning to both Alzheimer's disease (AD) and vascular dementia. With chronological age, the incidence of MCR increases and MCR cases exhibit a higher prevalence of age‐associated diseases. Biological age reflects an individual's accumulated physiological condition captured by changes at the epigenetic (DNA methylation) as well as proteomic levels (protein expression). Aging varies between individuals and at organ‐specific level within an individual. Our objectives were to (1) examine associations between biological age acceleration and MCR, (2) to identify organs that are aging at faster rate in MCR using novel organ‐specific clocks, and (3) impact of biological aging with mortality in MCR.

**Method:**

We measured 4,265 proteins in plasma collected at baseline in the LonGenity cohort (n=1,025, Age ≥65 years) – utilizing the SomaScan proteomic assay. Machine learning models were used to derive biological age predictors at the individual level from all the proteins. Organ‐specific ages for 11 organs were also computed from the organ‐specific plasma proteome. Participants were characterized as fast and slow agers based on these clocks. Age acceleration measures were regressed against prevalent and incident MCR using logistic and cox models, respectively. Associations with biological aging were validated using an epigenetic clock (DNAm GrimAge) in HRS (n=1,043).

**Result:**

Faster biological aging derived from the proteomic clock were associated with prevalent MCR as well as incident MCR. Prevalent MCR was most strongly associated with accelerated aging in muscles (p<0.001) and the heart (p<0.01). Brain, liver, kidney, and immune age acceleration was also nominally associated with prevalent MCR (p<0.05). Participants with both MCR and accelerated aging had the highest risk of mortality, as indicated by proteomic and epigenetic clocks in LonGenity (HR = 3.38) and HRS (HR = 2.47), respectively.

**Conclusion:**

Accelerated aging is a hallmark of MCR and have an impact on aging of multiple organs. Older individuals with MCR and accelerated aging are at highest risk of mortality. Biological age acceleration may be an important modifiable dementia risk factor.

